# A Solomon Link through an Interwoven Molecular Grid**

**DOI:** 10.1002/anie.201502095

**Published:** 2015-05-08

**Authors:** Jonathon E Beves, Jonathan J Danon, David A Leigh, Jean-François Lemonnier, Iñigo J Vitorica-Yrezabal

**Affiliations:** School of Chemistry, University of Edinburgh, The King's Buildings West Mains Road, Edinburgh, EH9 3JJ (UK); School of Chemistry, University of Manchester Oxford Road Manchester, M13 9PL (UK)

**Keywords:** catenanes, coordination chemistry, molecular grids, self-assembly, supramolecular chemistry

## Abstract

A molecular Solomon link was synthesized through the assembly of an interwoven molecular grid consisting of four bis(benzimidazolepyridyl)benzthiazolo[5,4-d]thiazole ligands and four zinc(II), iron(II), or cobalt(II) cations, followed by ring-closing olefin metathesis. NMR spectroscopy, mass spectrometry, and X-ray crystallography confirmed the doubly interlocked topology, and subsequent demetalation afforded the wholly organic Solomon link. The synthesis, in which each metal ion defines the crossing point of two ligand strands, suggests that interwoven molecular grids should be useful scaffolds for the rational construction of other topologically complex structures.

Knots and links are fundamental elements of structure, and have been exploited at the macroscopic level for millennia in the creation of tools (e.g. fishing nets) and materials (e.g. weaving), and in cultural and religious symbolism. Numerous biological examples illustrate how topological complexity at the molecular level can also confer significant physical and chemical properties (including improved strength, flexibility, stability, and dynamics).^1^ However, although the synthesis of the simplest [2]catenanes (singly interlocked rings; Hopf links^2^) has become almost routine,^3^ the rational synthesis of more complex topologies remains a very significant challenge.^4^ Of the higher-order molecular links prepared to date,^5^–^7^ about half were formed as unanticipated reaction products,^6^, 7b and there are currently only limited general strategies (e.g. via linear or cyclic helicates; Figure 1) for the rational construction of higher-order molecular links.

**Figure 1:**
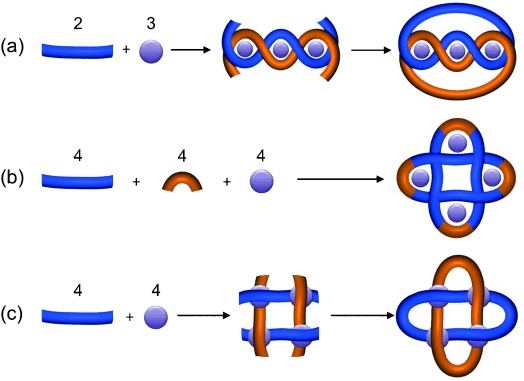
Metal-template strategies for the rational synthesis of molecular Solomon links: a) via a linear double helicate,5a,5b b) via a one-pot circular double helicate assembly,5d and c) via an interwoven molecular grid (this work).

The next simplest link topology after the Hopf link is the Solomon link (a ${4_1^2 }$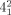 link in Alexander-Briggs notation^8^), a [2]catenane comprised of doubly interlocked rings.^2^ Sauvage reported the first synthesis5b of a molecular Solomon link by connecting the end groups of extended linear metal helicates (Figure 1 a), however the distance between the ligand strand termini makes this approach unsuitable for extrapolating to higher-order knots and links.^9^ A one-pot strategy using a cyclic helicate scaffold to bring the end groups into closer proximity has also been described (Figure 1 b),5d the ligand strands joined through reversible imine formation, which provides a mechanism through which connectivity errors can be corrected.^10^ Several other Solomon links with metal-bridged5c,5f, 6b,6c,6f or wholly organic6a,6d,6e frameworks have also been isolated. Here we report on the designed synthesis of a Solomon link utilizing an interwoven 2×2 molecular grid to create a crossing point at the site of each metal ion (Figure 1 c).

Molecular grids, which are polytopic ligands coordinated to two-dimensional arrays of metal ions, have attracted interest because of their potential magnetic and electronic properties.^11^ However, almost all the examples prepared to date consist of discrete layers of ligands that lie to each side of the tier of metal ions. Interwoven grids are much rarer,^12^ but the crossing of the ligand strands in such systems make them, in principle, suitable scaffolds for constructing various interlocked structures.4e Connecting the adjacent termini of parallel ligand strands in an interwoven 2×2 grid should afford a Solomon link (Figure 1 c).

In our efforts to produce such a system, we initially investigated functionalized analogues of ligands previously shown to form interwoven 2×2 grids, but the derivatives we prepared had poor solubility profiles as either the free ligand or as metal complexes. In the search for a new interwoven-grid-forming ligand, we identified **1** as a potential candidate (Scheme 1). Ligand **1** contains two, antiparallel, tridentate binding sites formed from a thiazolo[5,4-d]thiazole (TTZ) core connected on each side to pyridylbenzimidazole units.^13^ A bis(bipyridyl)TTZ compound has previously been synthesized as a potential bis-tridentate ligand,^14^ but its coordination chemistry has not been reported. Ligand **1** has terminal alkene chains appropriate for macrocyclization of pairs of **1** by olefin metathesis. The length of the alkyl linker was chosen so as to permit only connections between parallel ligand strands in the grid coordination complex (see the Supporting Information, Figure S12). Isopentyl groups were integrated into the benzimidazole framework to aid the solubility of the ligand and its complexes.

**Scheme 1:**
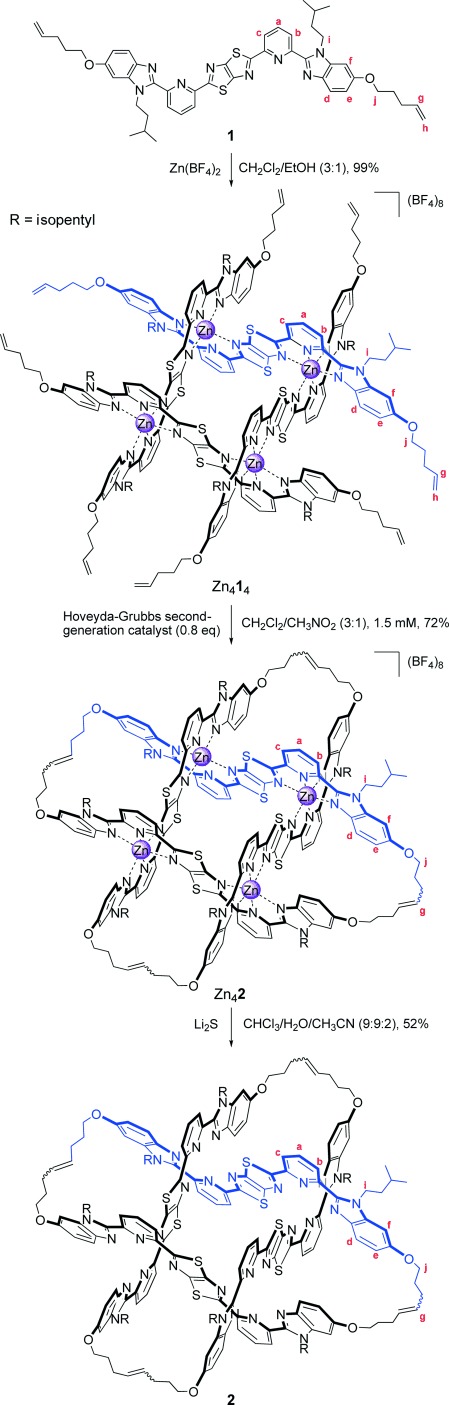
Synthesis of interwoven grid [Zn_4_1_4_](BF_4_)_8_ and Solomon links [Zn_4_2](BF_4_)_8_ and 2.

Addition of an equimolar amount of Zn(BF_4_)_2_ in ethanol to a suspension of **1** in dichloromethane resulted in the rapid solubilization of the ligand and the quantitative assembly of complex [Zn_4_**1**_4_](BF_4_)_8_ (Scheme 1). ^1^H NMR spectroscopy (Figure 2 b) showed the presence of a single species possessing the same two-fold symmetry as ligand **1**. Chemical shift changes of proton signals H^*a*^ (Δ*δ*=+0.92 ppm) and H^*d*^ (Δ*δ*=−1.20 ppm) indicated the formation of octahedral bis(tridentate) complexes consistent with a grid structure. The diastereotopic splitting of protons H^*i*^ confirmed that the two faces of each ligand were in different environments, an inherent property of an interwoven 2×2 grid.

**Figure 2:**
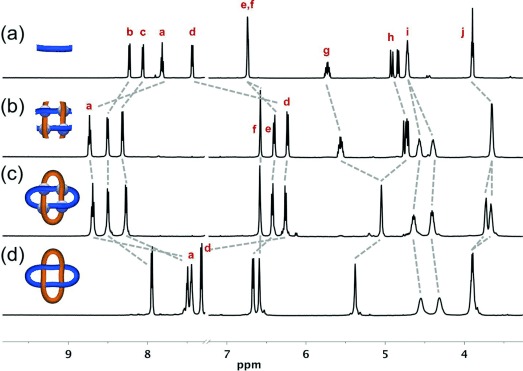
Partial ^1^H NMR spectra (2:1 CDCl_3_/CD_3_CN, 600 MHz, 298 K except for (d)) of a) ligand 1, b) grid complex [Zn_4_1_4_](BF_4_)_8_, c) Solomon link [Zn_4_2](BF_4_)_8_, and d) demetalated Solomon link 2 (325 K). Proton assignments refer to the labeling in Scheme 1.

Olefin metathesis using the Hoveyda–Grubbs second-generation catalyst^15^ (0.1 equiv per olefin) was carried out on [Zn_4_**1**_4_](BF_4_)_8_ in a 3:1 mixture of dichloromethane/nitromethane (1.5 mm grid concentration). After 12 h at room temperature, the catalyst was quenched, the solvent removed under reduced pressure and, after trituration with chloroform, a complex characterized as [Zn_4_**2**](BF_4_)_8_ was isolated in 72 % yield (92 % per bond). Multiply charged peaks corresponding to {[**2**](BF_4_)_*n*_}^(8−*n*)+^ (*n*=3–7) were observed by electrospray ionization mass spectrometry (ESI-MS), thus confirming the loss of four ethylene molecules from the open complex (Figure S1). The olefin region of the ^1^H NMR spectrum of [Zn_4_**2**](BF_4_)_8_ showed the terminal alkene signals (H^*g*^ and H^*h*^; Figure 2 b) of [Zn_4_**1**_4_](BF_4_)_8_ had been replaced by a signal corresponding to an internal alkene (Figure 2 c). Broadening of some of the alkyl linker resonances (when compared to the open precursor, Zn_4_**1**_4_) and the more pronounced diastereotopic splitting of proton H^*j*^ may be a result of the restricted rotation of the connecting chain in the cyclized complex.

Single crystals of [Zn_4_**2**](BF_4_)_8_ suitable for analysis by X-ray crystallography were obtained by slow vapor diffusion of diethyl ether into a saturated acetonitrile solution of the Solomon link. The solid-state structure^16^ (Figure 3) confirms the doubly entwined topology of the two 64-atom loops in [Zn_4_**2**]^8+^. The eight aromatic rings of each ligand strand are rendered virtually co-planar by coordination to octahedral zinc(II) cations in the interwoven 2×2 grid motif. In turn, the four metal centers form a near-perfect square (Zn-Zn-Zn angles of 90(1)°), thus ensuring that the two ligand strands of each macrocycle are close-to-parallel and orthogonal to those of the other macrocycle. Although the 600 MHz ^1^H NMR spectrum of [Zn_4_**2**](BF_4_)_8_ has only one signal for the alkene protons (H^*g*^, Figure 2 c), the X-ray crystal structure indicates that a mixture of *E* and *Z* olefins form during the olefin metathesis reaction. Six of the eight BF_4_^−^ anions were located in the structure (the remaining two are disordered over several sites), including one in the center of the Solomon link cavity (see the Supporting Information, Figures S10 and S11).

**Figure 3:**
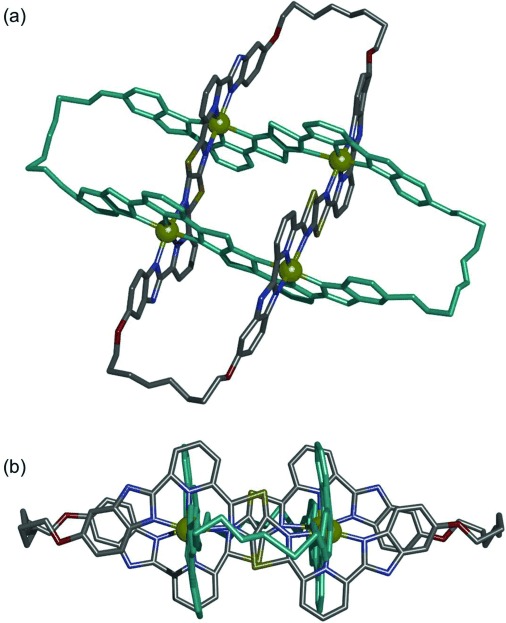
X-Ray crystal structure of Solomon link [Zn_4_2](BF_4_)_8_.^16^ Isopentyl groups, hydrogen atoms, solvent molecules, and counterions are omitted for clarity. One macrocycle is colored turquoise. Color code for the other macrocycle: C, grey; N, purple; O, red; S, gold; Zn, yellow. a) Viewed from above the plane of the zinc ions. b) Viewed from the side.

Demetalation of [Zn_4_**2**](BF_4_)_8_ using Li_2_S^17^ proceeded smoothly to afford the wholly organic Solomon link **2** in 52 % yield (Scheme 1). Significant chemical shifts of protons H^*a*^ (Δ*δ*=−1.25 ppm) and H^*d*^ (Δ*δ*=+1.06 ppm) in the ^1^H NMR spectrum were consistent with the ligands being metal-free (Figure 2 d). The protonated molecular ions [**2**⋅*n* H]^*n*+^ (*n*=1–4) were observed by ESI-MS (Figure 4 a), and on fragmentation of the [**2**⋅H]^+^ ion by tandem mass spectrometry, a singly charged species with half the molecular weight of **2** was evident; one constituent ring of the [2]catenane (Figure 4 b).^18^

**Figure 4:**
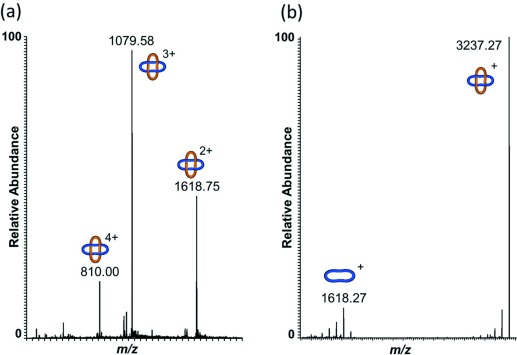
Partial ESI-MS spectra of a) demetalated Solomon link 2 and b) tandem MS fragmentation of the [2⋅H]^+^ ion to the constituent macrocycle. See the Supporting Information (Figure S6–S8) for further information.

We successfully repeated the assembly of the grid and Solomon link using either Fe(BF_4_)_2_ or Co(BF_4_)_2_ in place of Zn(BF_4_)_2_ and obtained the corresponding Solomon link complexes in each case (see the Supporting Information).^19^ Demetalation of these analogues afforded Solomon link **2** in similar yields to the zinc(II) template synthesis. While the signals in the ^1^H NMR spectrum of [Fe_4_**1**_4_](BF_4_)_8_ in CD_3_CN at room temperature are sharp, those of [Fe_4_**2**](BF_4_)_8_ are significantly broadened (Figure S2), thus indicating the presence of high-spin Fe^II^ species. Linking the ends of ligands in interwoven grids could prove useful for tuning systems that display spin-crossover behavior.^20^

In conclusion, ligand **1** can be quantitatively assembled into interwoven 2×2 molecular grids using several different octahedral first-row transition-metal ions. Ring-closing olefin metathesis covalently captures the entwined structure in high yield and subsequent removal of the cations affords the metal-free Solomon link. These results suggest that interwoven grids could form the basis of a general strategy for the rational synthesis of higher-order knots and links.4e The tolerance of the ligand system to different metal ions could potentially be used to impart magnetic,e electronic,11d,e, 20 or sensing^21^ properties to topologically complex molecular structures.

## Supporting Information

As a service to our authors and readers, this journal provides supporting information supplied by the authors. Such materials are peer reviewed and may be re-organized for online delivery, but are not copy-edited or typeset. Technical support issues arising from supporting information (other than missing files) should be addressed to the authors.

miscellaneous_information
