# Supplementary material for: A Solomon Link through an Interwoven Molecular Grid
Source: Angew Chem Int Ed Engl. 2015 May 8;54(26):7555–9. doi: 10.1002/anie.201502095 (PMC4479027; doi:10.1002/anie.201502095)
Supplement: Supplementary file 1 — miscellaneous_information [file anie0054-7555-sd1.pdf]

Angewandte Chemie
Eine Zeitschrift der Gesellschaft Deutscher Chemiker

Supporting Information

A Solomon Link through an Interwoven Molecular Grid**

Jonathon E. Beves, Jonathan J. Danon, David A. Leigh, Jean-François Lemonnier, and Iñigo J. Vitorica-Yrezabal*

anie_201502095_sm_miscellaneous_information.pdf

Table of Contents

1. General Experimental S2
2. Synthesis and Experimental S3
2.1. Synthesis of ligand **1** S3
2.2. Synthesis of molecular grids and Solomon links S6
3. X-ray Crystal Structure S16
4. Molecular modeling of Zn_4**2** S20
5. Supporting Information References S21

1. General Experimental

Unless stated otherwise, all reagents and solvents were purchased from Sigma Aldrich and used without further purification. 3-fluoro-4-nitrophenol was purchased from Fluorochem and was used without further purification. 6-(hydroxymethyl)picolinaldehyde was synthesized following literature procedure.[S1] Anhydrous THF and dichloromethane were obtained by passing the solvent (HPLC grade) through an activated alumina column on a JC Meyer solvent drying system. Anhydrous nitromethane was dried over molecular sieves and degassed by purging with nitrogen for 2 hours. Column chromatography was carried out using Silica 60Å (particle size 40-63 µm, Sigma Aldrich, UK) as the stationary phase, and TLC was performed on precoated silica gel plates (0.25 mm thick, 60 F_{254}, Merck, Germany) and observed under UV light. NMR spectra were recorded on a Bruker Avance 600 HD with an Oxford AS600 magnet (600 MHz cryoprobe). Chemical shifts are reported in parts per million (ppm) from low to high frequency and referenced to the residual solvent resonance. Coupling constants (*J*) are reported in hertz (Hz). Standard abbreviations indicating multiplicity were used as follows: s = singlet, d = doublet, t = triplet, dd = double doublet, q = quartet, m = multiplet, ddd = doublet of double doublets, ddt = doublet of double triplets. ^{1}H and ^{13}C NMR assignments were made using 2D-NMR methods (COSY, NOESY, HSQC, HMBC) and are unambiguous unless stated otherwise. Melting points (m.p.) were determined using a Buchi M-565 apparatus and are reported uncorrected. Low resolution ESI mass spectrometry was performed with a Thermo Scientific LCQ Fleet Ion Trap Mass Spectrometer or an Agilent Technologies 1200 LC system with 6130 single quadrupole MS detector. High-resolution mass spectrometry was carried out by the EPSRC National Mass Spectrometry Service Centre (Swansea, UK).

2. Synthesis and Experimental

2.1. Synthesis of ligand 1

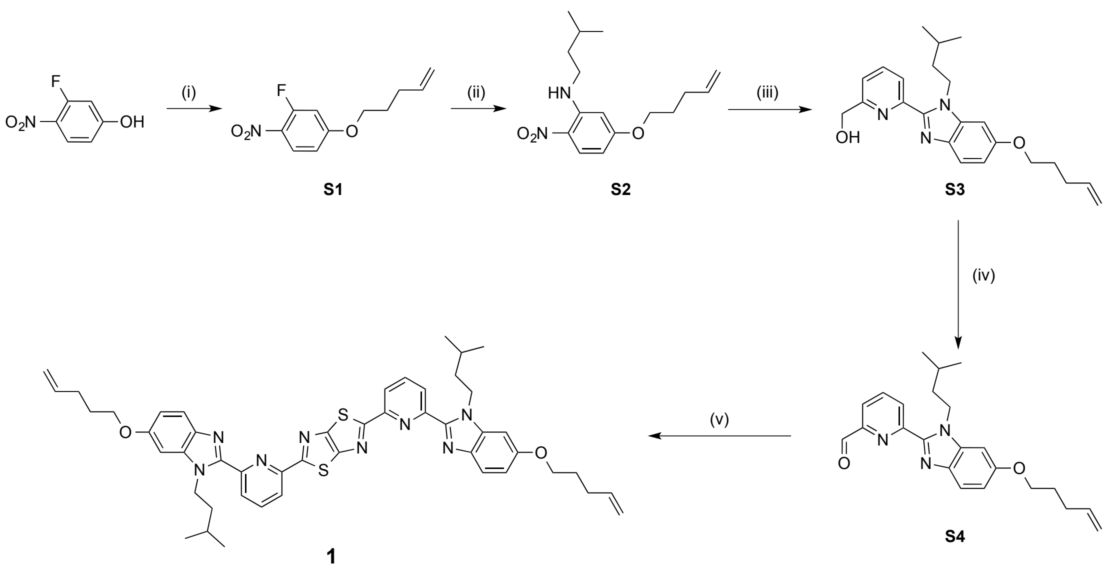

Scheme S1 Preparation of ligand **1**. Reaction conditions: (i) 5-Bromopent-1-ene, K_2CO_3, DMF, 60ºC, 89%. (ii) *iso*-pentylamine (2 eq), DMSO, 90ºC, quant. (iii) 1. 6-(hydroxymethyl)picolinaldehyde, water/2-methoxyethanol (1:4), 100ºC. 2. $Na_2S_2O_4$, 100ºC, 66%. (iv) MnO_2, $CHCl_3$, reflux, 84%. (v) Dithiooxamide (0.5 eq), DMF, 130ºC, 50%.

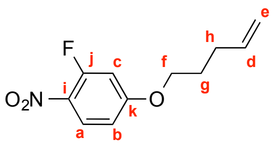

S1

A stirred solution of 3-fluoro-4-nitrophenol (4 g, 25.5 mmol), potassium carbonate (3.87 g, 28.0 mmol) and 5-bromo-1-pentene (4.17 g, 28.0 mmol) in DMF (25 mL) was heated at 60°C for 16 hours under an atmosphere of nitrogen. On cooling, the solvent was removed, and the residue partitioned between ethyl acetate (100 mL) and water (50 mL). The organic layer was then sequentially washed with 5% aqueous lithium chloride (3 x 50 mL), water (50 mL) and brine (50 mL). The organics were dried over $MgSO_4$ and the solvent removed under vacuum. The desired product **S1** was obtained as a brown oil (5.12 g, 89%) and was deemed sufficiently pure to use in the following step without further purification. ^{1}H NMR (600 MHz, $CDCl_3$) δ 8.07 (t, *J* = 9.2 Hz, H_a), 6.75 (dd, *J* = 9.2, 2.7 Hz, H_b), 6.71 (dd, *J* = 12.8, 2.7 Hz, H_c), 5.83 (ddt, *J* = 17.0, 10.2, 6.7 Hz, H_d), 5.06 (ddd, *J* = 17.0, 3.0, 1.4 Hz, $H_{e\text{-}cis}$), 5.03 (dd, *J* = 10.2, 1.2 Hz, $H_{e\text{-}trans}$), 4.04 (t, *J* = 6.4 Hz, H_f), 2.24 (q, *J* = 7.1 Hz, H_h), 1.97 – 1.88 (m, H_g). ^{13}C NMR (151 MHz, $CDCl_3$) δ 164.95 (d, *J* = 11.1 Hz, C_k), 157.63 (d, *J* = 264.9 Hz, C_j), 137.20 (C_d), 130.63 (d, *J* = 6.1 Hz, C_i), 128.01 (d, *J* = 1.1 Hz, C_a), 115.91 (C_e), 110.87 (d, *J* = 3.0 Hz, C_b), 103.63 (d, *J* = 24.1 Hz, C_c), 68.58 (C_f), 29.92 (C_h), 27.85 (C_g). HRESI-MS: *m/z* = 226.0868 $[M+H]^+$ (calcd. for $C_{11}H_{12}FNO_3$, 226.0874).

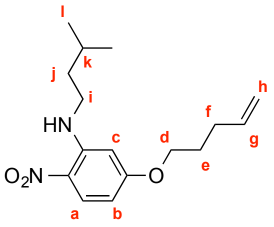

S2

To a stirred solution of **S1** (4.92 g, 21.9 mmol) in DMSO (25 mL) at 90°C was added isopentylamine (5.1 mL, 43.7 mmol). The resulting solution was stirred at the same temperature for 2 hours. On cooling, the solution was partitioned between water (50 mL) and diethyl ether (50 mL). The organic layer was dried over $MgSO_4$ and the solvent removed under vacuum. The desired product **S2** was obtained as a dark red oil (6.39 g, quant.) and was deemed sufficiently pure to use in the following step without further purification. ^{1}H NMR (600 MHz, $CDCl_3$) δ 8.25 (s, N-H), 8.12 (d, *J* = 9.5 Hz, H_a), 6.21 (dd, *J* = 9.5, 2.5 Hz, H_b), 6.12 (d, *J* = 2.5 Hz, H_c), 5.84 (ddt, *J* = 17.0, 10.2, 6.7 Hz, H_g), 5.07 (ddd, *J* = 17.0, 3.2, 1.5 Hz, $H_{h\text{-}cis}$), 5.02 (dd, *J* = 10.2, 1.5 Hz, $H_{h\text{-}trans}$), 4.03 (t, *J* = 6.4 Hz, H_d), 3.26 (tt, *J* = 7.2, 3.6 Hz, H_i), 2.24 (q, *J* = 6.9 Hz, H_f), 1.94 – 1.87 (m, H_e), 1.82 – 1.72 (m, H_k), 1.63 (dd, *J* = 14.4, 7.2 Hz, H_j), 0.98 (d, *J* = 6.6 Hz, H_l). ^{13}C NMR (151 MHz, $CDCl_3$) δ 165.60 (C-NO_2), 148.01 (C-NH), 137.49 (C_g), 129.38 (C_a), 126.41 (O-C_{Ar}), 115.70 (C_h), 104.85 (C_b), 95.78 (C_c), 67.69 (C_d), 41.37 (C_i), 37.76 (C_j), 30.06 (C_f), 28.25 (C_e), 26.07 (C_k), 22.61 (C_l). HRESI-MS: *m/z* = 293.1859 $[M+H]^+$ (calcd. for $C_{16}H_{24}N_2O_3$, 293.1860).

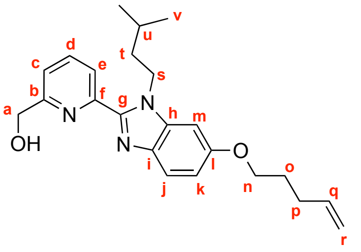

S3

6-(hydroxymethyl)picolinaldehyde (1.41 g, 10.26 mmol) was dissolved in a mixture of 2-methoxyethanol (30 mL) and water (7.7 mL). The solution was degassed with nitrogen bubbling for 1 hour and subsequently warmed to 60°C. **S2** (3.00 g, 10.26 mmol) was added, after which degassing was resumed for 15 minutes. The resulting biphasic solution was then stirred at 100°C for 1 hour, during which time the two layers became miscible. Finally, an excess of sodium dithionite (6 g) was added in one portion, yielding a bright yellow suspension. Stirring was continued under nitrogen at 100°C for 16 hours. After cooling to room temperature, water (30 mL) was added and most of the solvent was removed under vacuum. The residue was partitioned between CH_2Cl_2 (50 mL) and water (50 mL), and after separation the aqueous layer was extracted with more CH_2Cl_2 (2 x 50 mL). The combined organics were washed with water (50 mL) and brine (50 mL) before drying over Na_2SO_4 and removal of solvent under vacuum. Purification by flash column chromatography (SiO_2, CH_2Cl_2:MeOH, 99:1 to 97:3) yielded **S3** as a yellow solid (2.56 g, 66%). ^{1}H NMR (600 MHz, $CDCl_3$) δ 8.24 (d, *J* = 7.8 Hz, H_e), 7.82 (t, *J* = 7.8 Hz, H_d), 7.72 (d, *J* = 8.8 Hz, H_j), 7.34 (d, *J* = 7.8 Hz, H_c), 6.97 (dd, *J* = 8.8, 2.3 Hz, H_k), 6.85 (d, *J* = 2.3 Hz, H_m), 5.89 (ddt, *J* = 17.0, 10.2, 6.7 Hz, H_q), 5.10 (ddd, *J* = 17.0, 3.3, 1.6 Hz, $H_{r\text{-}cis}$), 5.03 (dd, *J* = 10.2, 1.3 Hz, $H_{r\text{-}trans}$), 4.85 (s, H_a), 4.74 – 4.63 (m, H_s), 4.07 (t, *J* = 6.4 Hz, H_n), 3.48 (s, O-H), 2.30 (dd, *J* = 14.6, 7.0 Hz, H_p), 2.02 – 1.90 (m, H_o), 1.79 – 1.73 (m, H_t), 1.73 – 1.66 (m, H_u), 0.96 (d, *J* = 6.5 Hz, H_v). ^{13}C NMR

(151 MHz, $CDCl_3$) δ 158.75 (C_b), 156.73 (C_l), 148.86 (C_f), 148.71 (C_g), 137.84 (C_d), 137.68 (C_q), 136.95 (C_i), 123.32 (C_e), 120.46 (C_j), 120.42 (C_c), 115.29 (C_r), 112.83 (C_k), 112.81 (C_h), 94.35 (C_m), 67.98 (C_n), 64.62 (C_a), 44.22 (C_s), 38.72 (C_t), 30.20 (C_p), 28.52 (C_o), 26.33 (C_u), 22.53 (C_v). HRESI-MS: *m/z* = 380.2332 $[M+H]^+$ (calcd. for $C_{23}H_{29}N_3O_2$, 380.2333). m.p. 78-80ºC.

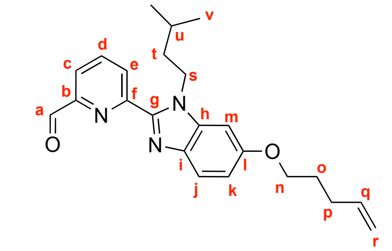

S4

To a solution of **S3** (150 mg, 0.40 mmol) in chloroform (10 mL) was added MnO_2 (348 mg, 4.00 mmol). The suspension was stirred at reflux for 16 hours before cooling, filtering over celite and removal of solvent under vacuum. The desired product **S4** was obtained as a dark brown oil (125 mg, 84%) and was deemed sufficiently pure to use in the following step without further purification. ^1H NMR (600 MHz, $CDCl_3$) δ 10.15 (s, H_a), 8.66 (s, H_d), 7.99 (m, H_{c+e}), 7.72 (s, H_j), 6.99 (s, H_k), 6.88 (s, H_m), 5.90 (ddt, *J* = 13.1, 10.2, 6.5 Hz, H_q), 5.10 (d, *J* = 16.9 Hz, $H_{r\text{-}cis}$), 5.03 (d, *J* = 10.2 Hz, $H_{r\text{-}trans}$), 4.85 (s, H_s), 4.09 (s, H_n), 2.31 (dd, *J* = 13.7, 6.8 Hz, H_p), 1.96 (s, H_o), 1.85 (s, H_t), 1.78 (s, H_u), 1.02 (d, *J* = 6.2 Hz, H_v). ^{13}C NMR (151 MHz, $CDCl_3$) δ 192.83 (C_a), 156.95 (C_l), 151.90 (C_b), 151.46 (C_f), 147.77 (C_g), 137.93 ($C_{c/e}$), 137.89 (C_q), 137.48 (C_i), 137.35 (C_h), 128.43 (C_d), 121.21 ($C_{c/e}$), 120.90 (C_j), 115.34 (C_r), 112.99 (C_k), 94.22 (C_m), 67.99 (C_n), 44.50 (C_s), 38.82 (C_t), 30.25 (C_p), 28.57 (C_o), 26.57 (C_u), 22.70 (C_v). HRESI-MS: *m/z* = 378.2175 $[M+H]^+$ (calcd. for $C_{23}H_{27}N_3O_2$, 378.2176).

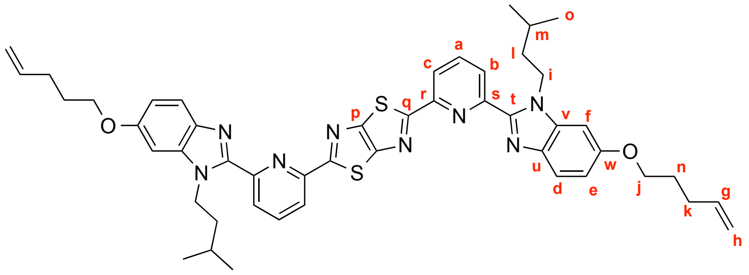

1

A solution of **S4** (125 mg, 0.33 mmol) and dithiooxamide (19.9 mg, 0.16 mmol) in DMF (4 mL) was heated at 130°C for 16 hours. After cooling, the solvent was removed under vacuum and the residue resuspended in methanol with agitation *via* an ultrasonic bath for 10 minutes. Vacuum filtration afforded **1** (70 mg, 50%) as an orange powder, which required no further purification. ^1H NMR (600 MHz, $CDCl_3$) δ 8.52 (d, *J* = 7.7 Hz, H_b), 8.30 (d, *J* = 7.7 Hz, H_c), 8.00 (t, *J* = 7.7 Hz, H_a), 7.74 (d, *J* = 8.7 Hz, H_d), 6.99 (dd, *J* = 8.7, 1.6 Hz, H_e), 6.92 (d, *J* = 1.6 Hz, H_f), 5.91 (ddt, *J* = 17.0, 10.2, 6.6 Hz, H_g), 5.11 (d, *J* = 17.0 Hz, $H_{h\text{-}cis}$), 5.04 (d, *J* = 10.2 Hz, $H_{h\text{-}trans}$), 5.00 – 4.91 (m, H_i), 4.10 (t, *J* = 6.4 Hz, H_j), 2.32 (q, *J* = 7.1 Hz, H_k), 2.01 – 1.94 (m, H_n), 1.89 – 1.78 (m, H_{l+m}), 1.01 (d, *J* = 6.2 Hz, H_o). ^{13}C NMR (151 MHz, $CDCl_3$) δ 170.59 (C_q), 157.15 (C_w), 153.38 (C_p), 150.42 (C_r), 147.58 (C_t), 138.30 (C_a), 137.93 (C_g), 137.24 (C_v), 134.26 (C_u), 126.29 (C_b), 120.67 (C_d), 120.09 (C_c), 115.45 (C_h), 113.54 (C_e), 94.38 (C_f), 68.09 (C_i), 44.29 (C_j), 39.14 (C_l), 30.33 (C_k), 28.61 (C_n), 26.28 (C_m), 22.92 (C_o). C_s was not

observed. HRESI-MS: *m/z* = 837.3726 $[M+H]^+$ (calcd. for $C_{48}H_{52}N_8O_2S_2$, 837.3727). m.p. 248-252°C.

2.2. Synthesis of molecular grids and Solomon links

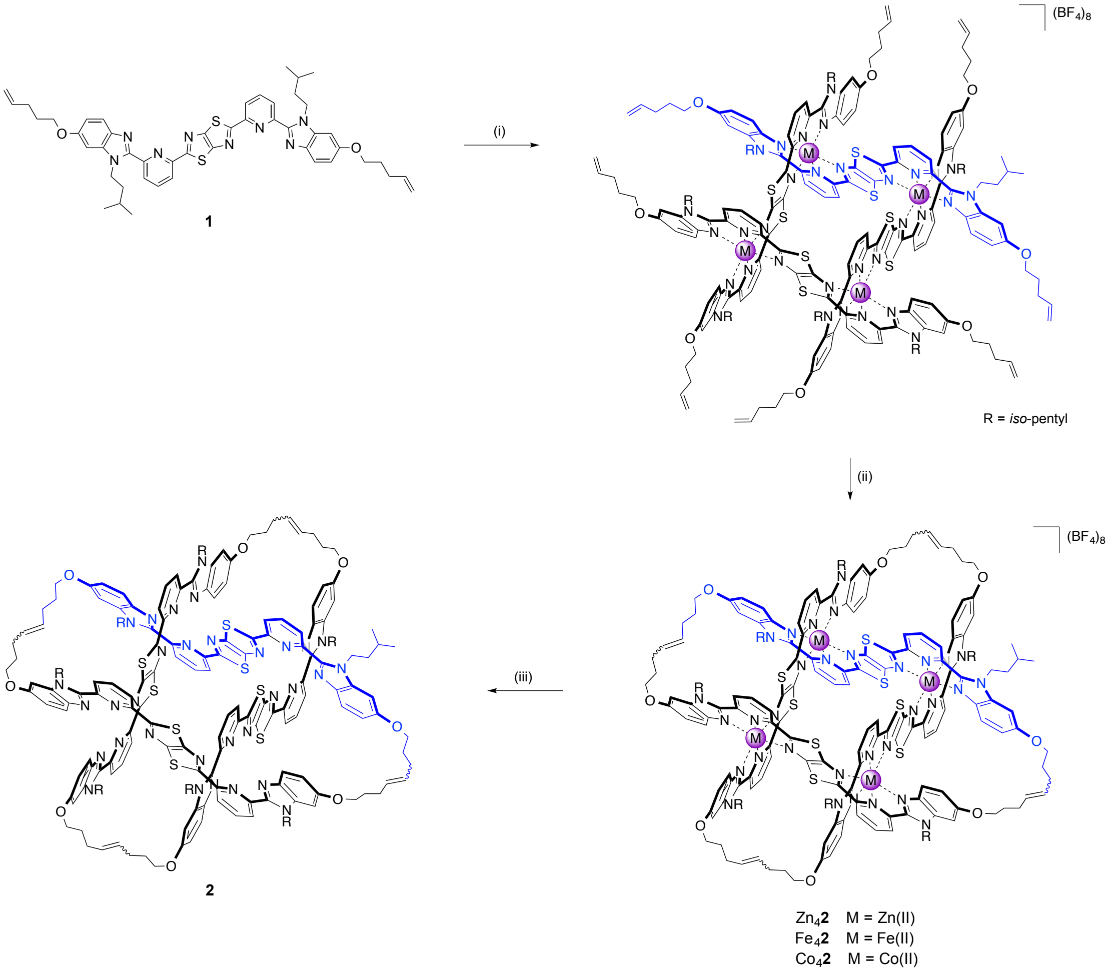

Scheme S2 Preparation of interwoven supramolecular grids and molecular Solomon links. Reaction conditions: (i) $M(BF_4)_2$, M = Zn(II), Fe(II) and Co(II), 3:1 CH_2Cl_2/EtOH, all quasi-quantitative yield. (ii) Hoveyda-Grubbs second generation catalyst (0.8 eq), 1.5 mM in 3:1 CH_2Cl_2/CH_3NO_2 (Zn$_4$**2**, 72%) or CH_2Cl_2 (Fe$_4$**2** 90%, Co$_4$**2** 71%). (iii) Zn$_4$**2**: Li_2S (10 eq), $CHCl_3$/water/CH_3CN (9:9:2), 52%. Fe$_4$**2**: TBACN (30 eq), CH_3CN, 46%. Co$_4$**2**: Na_2EDTA, CH_3CN/H_2O (1:1), 55 ºC, 56%.

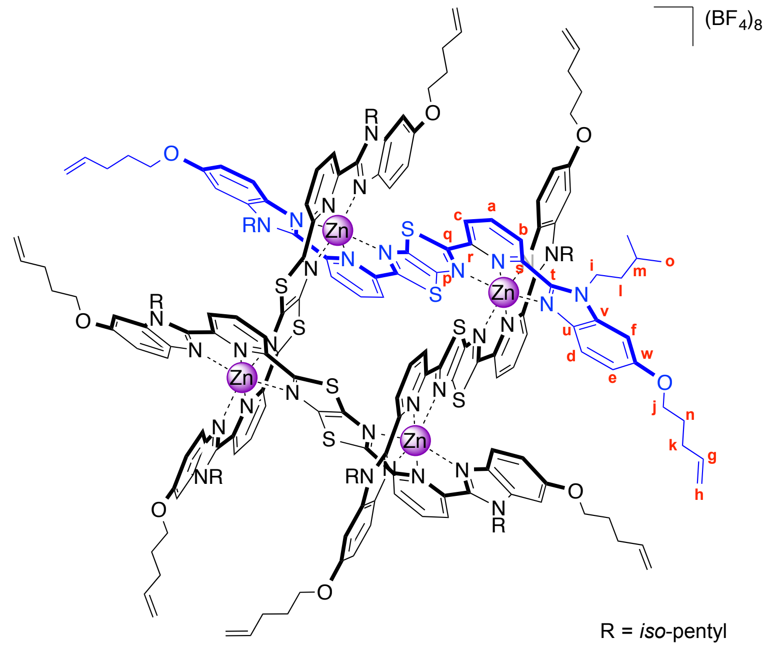

[Zn$_4$1$_4$](BF$_4$)$_8$

To a stirring suspension of **1** (11.8 mg, 14.1 µmol) in CH_2Cl_2 (1.0 mL) at room temperature was added a solution of zinc (II) tetrafluoroborate hydrate (5.0 mg, 14.1 µmol) in ethanol (0.3 mL). Within a minute the yellow suspension had become a clear yellow solution. Stirring was continued for one hour, after which the solvents were removed under vacuum. The crude product was dissolved in acetonitrile before passing over a small plug of celite. Removal of the solvent under vacuum afforded the desired complex [Zn$_4$**1**$_4$](BF$_4$)$_8$ (15.1 mg, 99%) as a yellow solid. ^{1}H NMR (600 MHz, CD_3CN) δ 8.87 (t, *J* = 8.2 Hz, H$_a$), 8.67 (d, *J* = 8.3 Hz, H$_b$), 8.34 (d, *J* = 8.0 Hz, H$_c$), 6.88 (d, *J* = 1.9 Hz, H$_f$), 6.57 (dd, *J* = 9.3, 2.0 Hz, H$_e$), 6.48 (d, *J* = 9.3 Hz, H$_d$), 5.78 (ddt, *J* = 17.0, 10.2, 6.7 Hz, H$_g$), 4.94 (dd, *J* = 17.2, 1.7 Hz, H$_{h\text{-}cis}$), 4.90 (d, *J* = 10.2 Hz, H$_{h\text{-}trans}$), 4.67 (t, *J* = 8.1 Hz, H$_i$), 3.95 – 3.75 (m, H$_j$), 2.14 – 2.06 (m, H$_k$), 1.88 – 1.77 (m, H$_l$), 1.77 – 1.64 (m, H$_{m+n}$), 0.97 (dd, *J* = 26.6, 6.6 Hz, H$_o$). ^{13}C NMR (151 MHz, CD_3CN) δ 167.10 (C$_q$), 159.44 (C$_w$), 151.59 (C$_p$), 147.79 (C$_a$), 145.97 (C$_r$), 145.73 (C$_t$), 144.14 (C$_s$), 139.08 (C$_v$), 138.86 (C$_g$), 132.67 (C$_u$), 127.13 (C$_b$), 125.45 (C$_c$), 119.45 (C$_f$), 117.95 (C$_e$), 115.60 (C$_h$), 95.23 (C$_d$), 68.91 (C$_j$), 45.59 (C$_i$), 38.98 (C$_l$), 30.58 (C$_k$), 28.80 (C$_n$), 26.73 (C$_m$), 22.76 + 22.55 (C$_o$). Analysis (% calcd, % found) for $C_{192}H_{208}B_8F_{32}N_{32}O_8S_8Zn_4$ + Et_2O: C (53.77, 53.56), H (5.02, 5.24), N (10.24, 10.19), S (5.86, 6.33), Zn (5.97, 5.96).

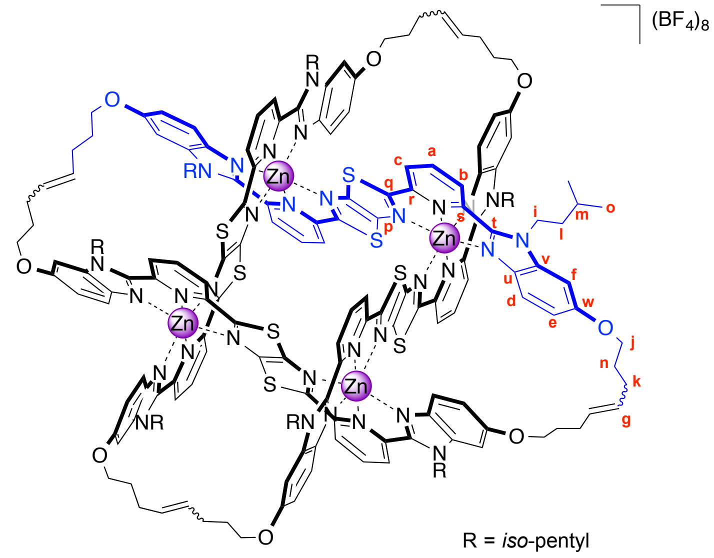

Zn$_4$**2**

[Zn$_4$**1**$_4$](BF$_4$)$_8$ (256 mg, 60 µmol) was dissolved in anhydrous and degassed nitromethane (10 mL) in a oven-dried flask equipped with magnetic stirrer bar under argon. Hoveyda-Grubbs 2nd generation catalyst (30 mg, 48 µmol) was added as a solution in anhydrous, degassed CH_2Cl_2 (30 mL) and the reaction mixture stirred at room temperature for 24 hours. Ethylvinyl ether was added to the reaction, followed by stirring for a further 30 minutes, before removing the solvent under reduced pressure. The resulting solid was sonnicated in chloroform (25 mL) for 15 minutes, filtered onto celite, washed with an excess of fresh chloroform and finally redissolved in acetonitrile. Removal of the solvent under vacuum afforded the product [Zn$_4$**2**](BF$_4$)$_8$ (180 mg, 72%) as a yellow solid. ^{1}H NMR (600 MHz, CD_3CN) δ 8.86 (t, *J* = 8.2 Hz, H_a), 8.68 (d, *J* = 8.3 Hz, H_b), 8.40 (d, *J* = 8.0 Hz, H_c), 6.86 (d, *J* = 1.5 Hz, H_f), 6.56 (dd, *J* = 9.2, 1.9 Hz, H_e), 6.45 (d, *J* = 9.2 Hz, H_d), 5.27 – 5.24 (m, H_g), 4.69 (t, *J* = 7.8 Hz, H_i)), 3.94 – 3.83 (m, H_j), 2.10 – 2.02 (m, H_k), 1.86 – 1.76 (m, H_{m+n}), 1.62 – 1.54 (m, H_l), 0.93 (dd, *J* = 37.4, 6.6 Hz, H_o). ^{13}C NMR (151 MHz, CD_3CN) δ 166.48 (C_q), 159.17 (C_w), 151.56 (C_p), 147.87 (C_a), 145.82 (C_r), 145.60 (C_t), 144.12 (C_s), 139.11 (C_v), 132.64 (C_u), 130.65 (C_g), 127.11 (C_b), 125.39 (C_c), 119.64 (C_f), 118.79 (C_e), 94.02 (C_d), 66.69 (C_j), 45.42 (C_i), 38.99 (C_l), 28.68 (C_k), 28.32 (C_n), 26.55 (C_m), 22.90 + 22.48 (C_o). HRESI-MS: *m/z* = 751.6124 [M–3(BF$_4$)]$^{5+}$ (calcd. for $C_{184}H_{192}Zn_4N_{32}O_8S_8(BF_4)_3$: 751.6117).

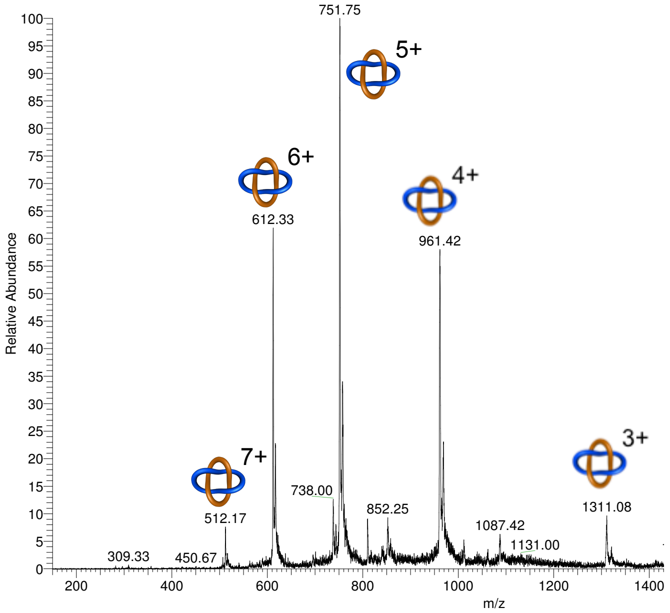

Figure S1 LRESI-MS spectrum of [Zn$_4$**2**](BF$_4$)$_8$.

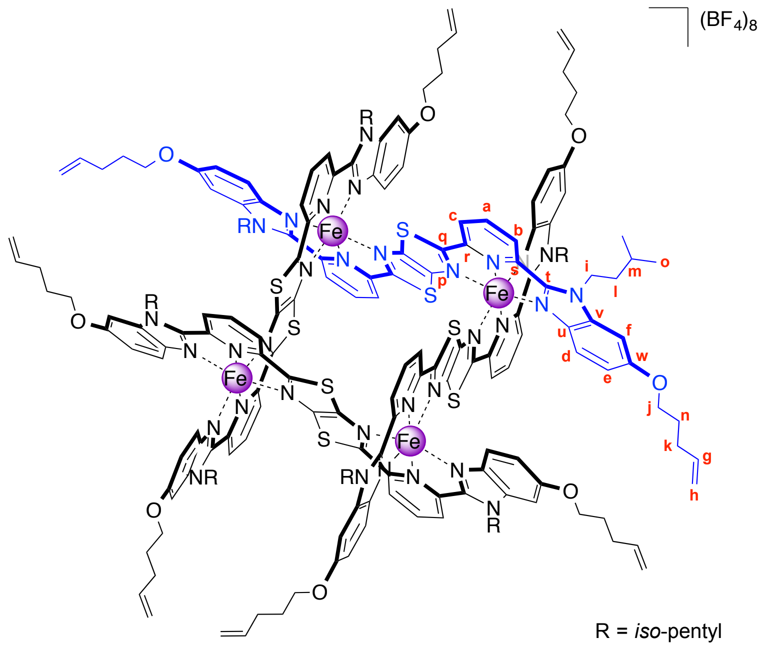

[$Fe_4$1$_4$](BF_4)$_8$

To a stirring suspension of **1** (30.0 mg, 36 µmol) in CH_2Cl_2 (3 mL) at room temperature was added a solution of iron (II) tetrafluoroborate hydrate (12.1 mg, 36.0 µmol) in ethanol (0.7 mL). Within a minute the yellow suspension had become an intensely dark green solution. Stirring was continued for one hour, after which the solvents were removed under vacuum. The residue was redissolved in acetonitrile before passing over a small plug of celite. Removal of the solvent under vacuum afforded the desired complex [Fe_4**1**$_4$](BF_4)$_8$ (36.3 mg, 96%) as a dark green solid. ^{1}H NMR (600 MHz, CD_3CN) δ 9.38 (s, H_b), 9.30 (d, J = 6.1 Hz, H_c), 9.08 (t, J = 8.1 Hz, H_a), 6.77 (s, H_f), 6.35 (d, J = 8.6 Hz, H_e), 5.73 (ddt, J = 13.4, 10.2, 6.6 Hz, H_g), 5.05 (d, J = 7.3 Hz, H_d), 4.94 – 4.83 (m, H_h), 4.81 – 4.64 (m, H_i), 3.79 – 3.62 (m, H_j), 2.05 – 1.98 (m, H_k), 1.64 (dt, J = 13.5, 6.6 Hz, H_n), 1.61– 1.53 (m, H_l), 1.34 – 1.23 (m, H_m), 0.61 (dd, J = 8.8, 7.0 Hz, H_o). ^{13}C NMR (151 MHz, CD_3CN) δ 158.99 (C_w), 149.31 (C_t), 143.28 (C_a), 138.77 (C_g), 128.17 (C_{b+c}), 117.98 (C_e), 116.87 (C_d), 115.56 (C_h), 96.62 (C_f), 68.80 (C_j), 45.61 (C_i), 39.69 (C_l), 30.49 (C_k), 28.66 (C_n), 26.36 (C_m), 22.63 + 22.50 (C_o). Quaternary carbons p, q, r, s, u and v were not observed. Analysis (% calcd, % found) for $C_{192}H_{208}B_8F_{32}N_{32}O_8S_8Fe_4$ + 2H_2O: C (53.59, 53.34), H (4.97, 5.11), N (10.41, 10.26), S (5.94, 6.46), Fe (5.19, 4.68).

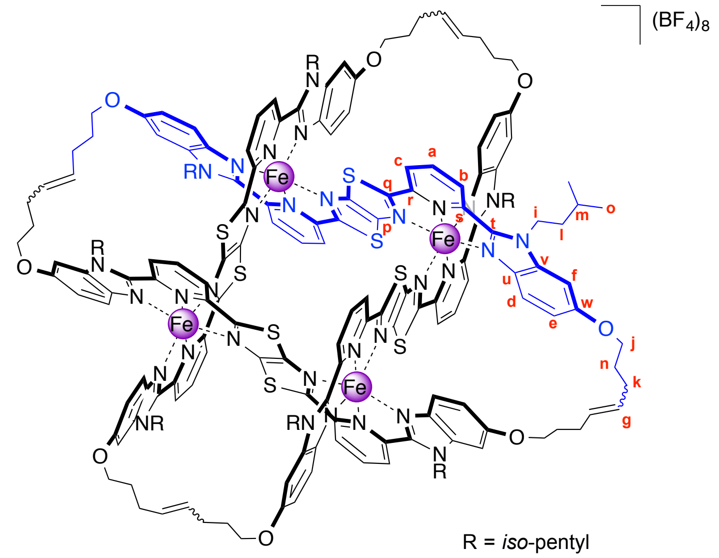

Fe$_4$**2**

To a dry flask under nitrogen was added [Fe$_4$**1**$_4$](BF$_4$)$_8$ (11.1 mg, 2.6 μmol) and Hoveyda-Grubbs 2nd generation catalyst (1.3 mg, 2.1 μmol, 0.8 eq) as solids. Anhydrous, degassed CH_2Cl_2 (2.6 mL) was added and the reaction mixture stirred at room temperature for 48 hours. Ethylvinyl ether was added to the reaction, followed by stirring for a further 30 minutes, before removing the solvent under reduced pressure. The resulting solid was sonnicated in $CHCl_3$ (10 mL) for 15 minutes, filtered onto celite, washed with an excess of fresh chloroform and finally redissolved in CH_2Cl_2. Removal of the solvent under vacuum afforded the product [Fe$_4$**2**](BF$_4$)$_8$ (10.0 mg, 93%) as a dark green solid. HRESI-MS: *m/z* = 744.0176 [M–3(BF$_4$)]$^{5+}$ (calcd. for $C_{184}H_{192}Fe_4N_{32}O_8S_8(BF_4)_3$: 744.0180).
As can be seen in Figure S2, the broad ^{1}H NMR of [Fe$_4$**2**](BF$_4$)$_8$ indicates some amount of high-spin Fe(II) complex. As such, the peaks could not be unambiguously assigned and ^{13}C NMR data were not obtained.

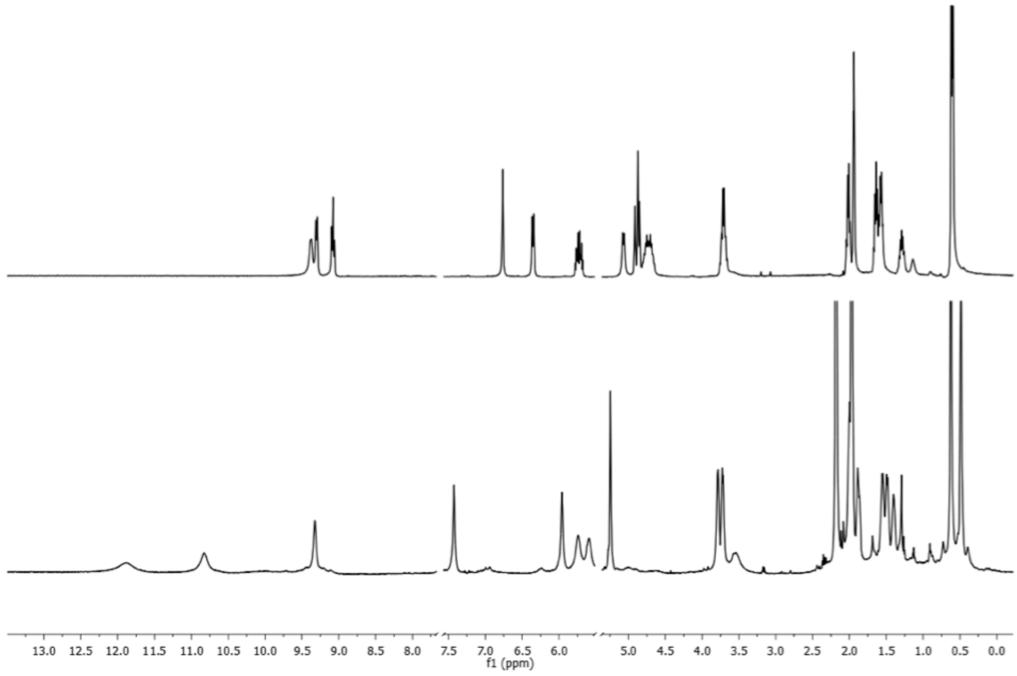

Figure S2 ^{1}H NMR (600 MHz, CD_3CN, 298 K) spectrum of [Fe$_4$**1**$_4$](BF$_4$)$_8$ (top) and [Fe$_4$**2**](BF$_4$)$_8$ (bottom).

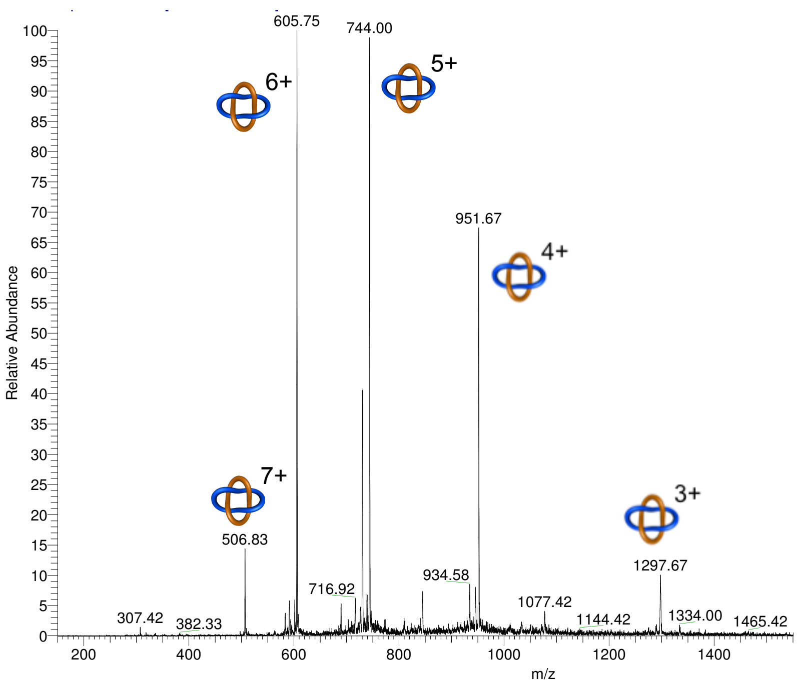

Figure S2 LRESI-MS spectrum of $[Fe_4\mathbf{2}](BF_4)_8$.

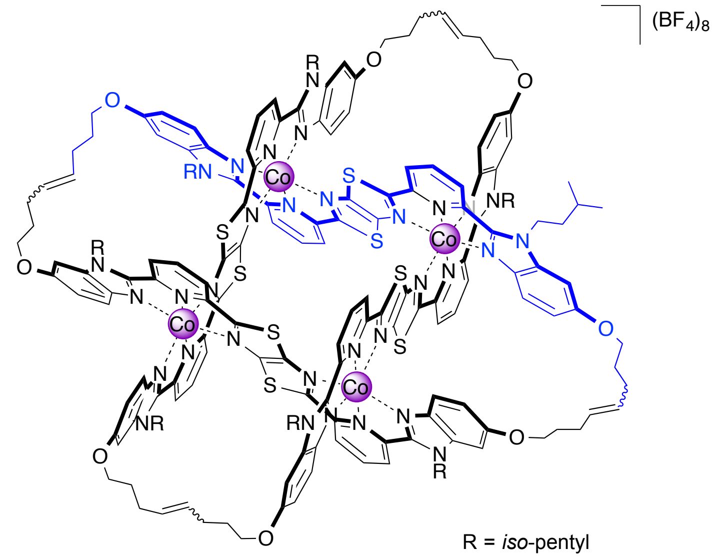

Co$_4$**2**

To a stirring suspension of **1** (17.8 mg, 21.3 μmol) in CH_2Cl_2 (3 mL) at room temperature was added a solution of cobalt (II) tetrafluoroborate hydrate (7.2 mg, 21.3 μmol) in ethanol (0.9 mL). Within seconds the yellow suspension had become a slightly turbid red solution. Stirring was continued for one hour, after which the solvents were removed under vacuum. The residue was redissolved in acetonitrile before passing over a small plug of celite. The solvent was removed under vacuum and the flask inerted with nitrogen. Addition of Hoveyda-Grubbs 2nd generation catalyst (1.3 mg, 2.1 μmol, 0.8 eq) as a solid against a flow of nitrogen was followed by addition of anhydrous, degassed CH_2Cl_2 (5.0 mL). The reaction mixture was stirred at room temperature for 48 hours. The catalyst was quenched by addition of ethylvinyl ether, before removal of all solvents under reduced pressure. The resulting solid was sonnicated in $CHCl_3$ (10 mL) for 15 minutes, filtered over celite, washed with an excess of fresh chloroform and finally redissolved in CH_3CN. Removal of the solvent under vacuum afforded the product [Co$_4$**2**]$(BF_4)_8$ (17 mg, 71%) as a red solid.

The ^{1}H NMR spectrum between 0 and 55 ppm showed the correct number of peaks for complex [Co$_4$**2**]$(BF_4)_8$, but due to the paramagnetic nature of cobalt(II) complexes, these were not unambiguously assignable. Nevertheless, subsequent demetallation of [Co$_4$**2**]$(BF_4)_8$ demonstrated that the desired Solomon link had been successfully synthesised.

HRESI-MS: *m/z* = 746.4165 $[M{-}3(BF_4)]^{5+}$ (calcd. for $C_{184}H_{192}Co_4N_{32}O_8S_8(BF_4)_3$: 746.4165).

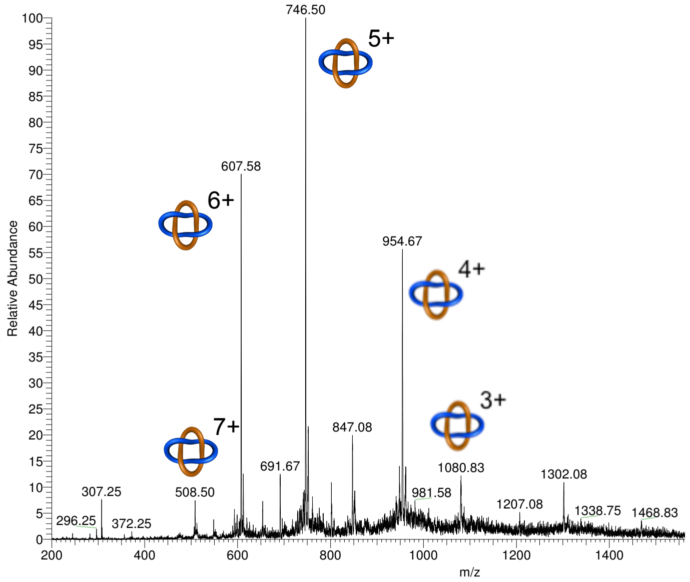

Figure S3 LRESI-MS spectrum of [Co$_4$**2**]$(BF_4)_8$.

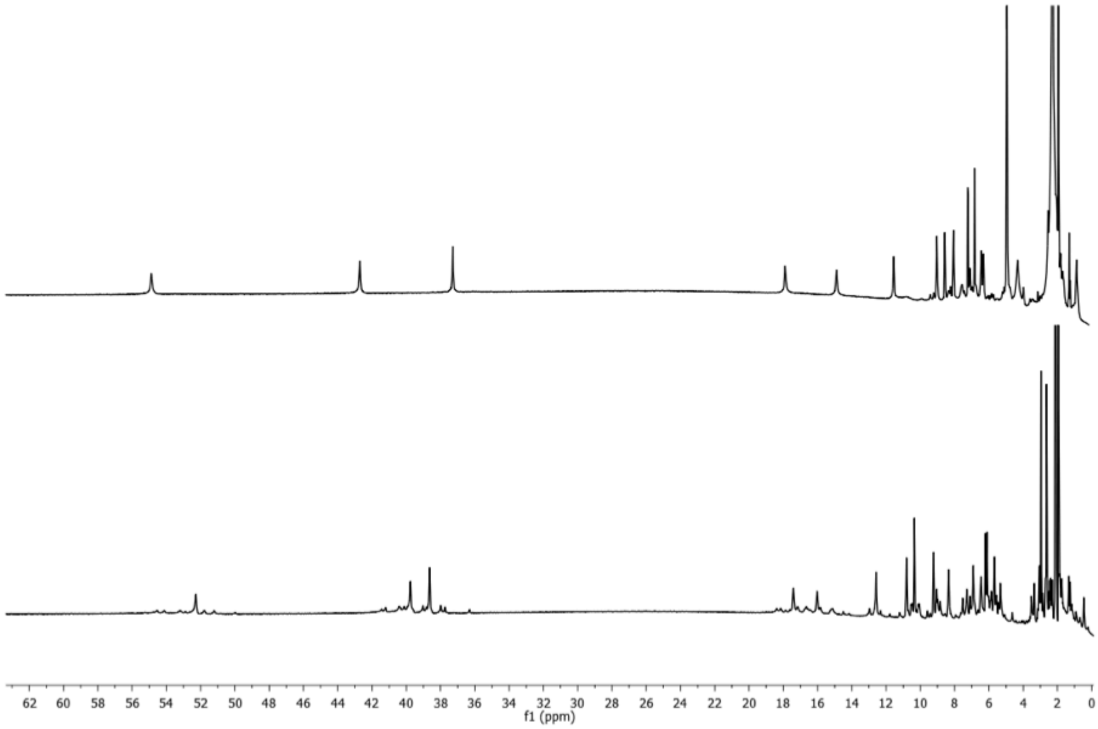

Figure S5 ^{1}H NMR (600 MHz, CD_3CN, 298 K) spectrum of $[Co_4\mathbf{1}_4](BF_4)_8$ (top) and $[Co_4\mathbf{2}](BF_4)_8$ (bottom).

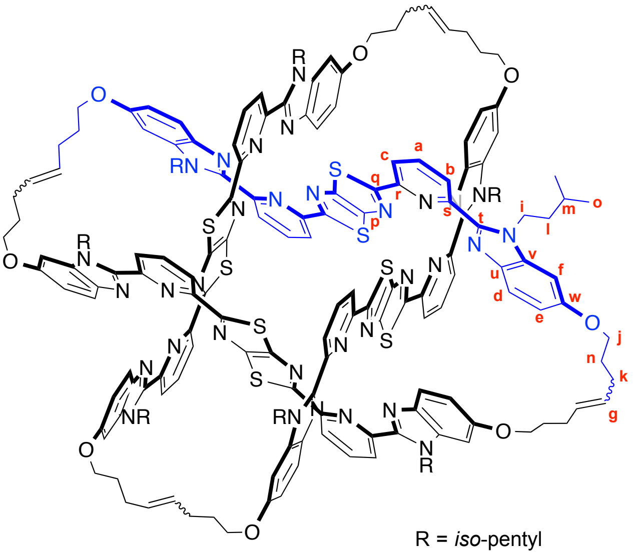

2

<u>Method A</u>

To a biphasic solution of $[Zn_4\mathbf{2}](BF_4)_8$ (20.0 mg, 4.8 µmol) in 9:9:2 $CHCl_3$/water/CH_3CN (5 mL) was added Li_2S (20 mg, large excess). The mixture was stirred vigorously for 1 hour then diluted with $CHCl_3$ (20 mL) and water (20 mL). The phases were separated and the aqueous layer was extracted with 2 x $CHCl_3$ (20 mL). The combined organic layers were dried over Na_2SO_4 and the solvent removed under vacuum. Purification by preparative thin layer chromatography using 96:3:1 CH_2Cl_2/MeOH/Et_3N as eluent afforded **2** (8.0 mg, 52%) as a bright yellow powder.

Method B

To a solution of [Fe_4**2**](BF_4)$_8$ (4.5 mg, 1.1 μmol) in acetonitrile (1 mL) was added tetrabutylammonium cyanide (8.7 mg, 30 eq). The mixture was stirred at room temperature for 16 hours, then partially purified by preparative thin layer chromatography using 96:3:1 CH_2Cl_2/MeOH/Et_3N as eluent. Residual tetrabutylammonium salts were removed by precipitation with methanol, affording **2** (1.6 mg, 46%) as a bright yellow solid.

Method C

To a solution of [Co_4**2**](BF_4)$_8$ (8.0 mg, 1.9 μmol) in acetonitrile (1 mL) was added saturated aqueous Na_2H_2EDTA (1 mL). The mixture was stirred at 55ºC for 2 hours before cooling and removal of all solvent under vacuum. The residue was diluted with $CHCl_3$ (20 mL) and water (20 mL). The phases were separated and the aqueous layer extracted with 3 x $CHCl_3$ (20 mL). The combined organic layers were dried over Na_2SO_4 and the solvent removed under vacuum. Purification by preparative thin layer chromatography using 96:3:1 CH_2Cl_2/MeOH/Et_3N as eluent afforded **2** (3.5 mg, 56%) as a bright yellow powder.

^{1}H NMR (600 MHz, 2:1 $CDCl_3$/CD_3CN, 325 K) δ 7.93 (d, *J* = 7.4 Hz, H_b), 7.49 (d, *J* = 7.4 Hz, H_c), 7.43 (t, *J* = 7.1 Hz, H_a), 7.30 (d, *J* = 8.6 Hz, H_d), 6.65 (d, *J* = 8.6 Hz, H_e), 6.58 (s, H_f), 5.37 (m, H_g), 4.54 (s, H_i), 4.30 (s, $H_{i'}$), 3.97 – 3.77 (m, H_j), 2.04 (m, H_k), 1.73 – 1.65 (m, H_n), 1.20 – 1.02 (m, H_{l+m}), 0.45 (t, *J* = 6.5 Hz, H_o). ^{13}C NMR (151 MHz, 2:1 $CDCl_3$/CD_3CN, 325 K) δ 169.33 (C_q), 155.87 (C_w), 152.29 (C_p), 149.92 (C_r), 149.16 (C_s), 147.57 (C_t), 137.07 (C_a), 136.63 (C_u), 136.56 (C_v), 129.65 (C_g), 124.62 (C_b), 119.95 (C_d), 118.73 (C_c), 112.20 (C_e), 94.14 (C_f), 67.21 (C_i), 42.91 (C_j), 38.47 (C_l), 29.05 (C_k), 28.28 + 28.22 (C_n), 24.97 (C_m), 21.93 + 21.76 (C_o). HRESI-MS: *m/z* = 1618.6785 $[M+2H]^{2+}$ (calcd. for $C_{184}H_{194}N_{32}O_8S_8$, 1618.6782).

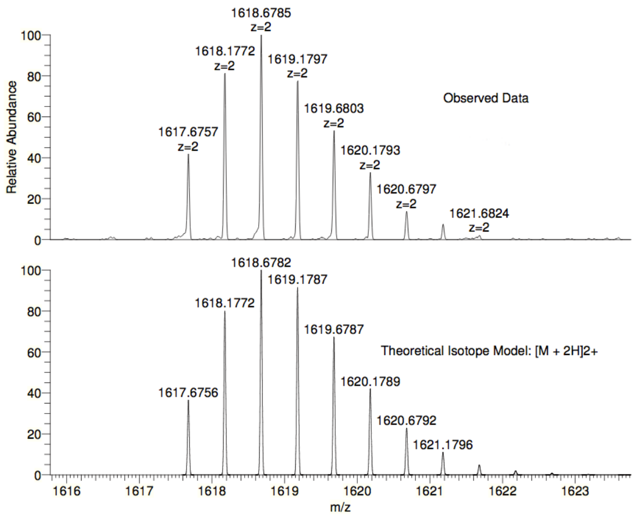

Figure S6 HRESI-MS analysis of **2**, showing excellent correlation between observed (top) and calculated (bottom) isotopic distributions of the doubly-charged $[\mathbf{2}{\bullet}2H]^{2+}$ ion.

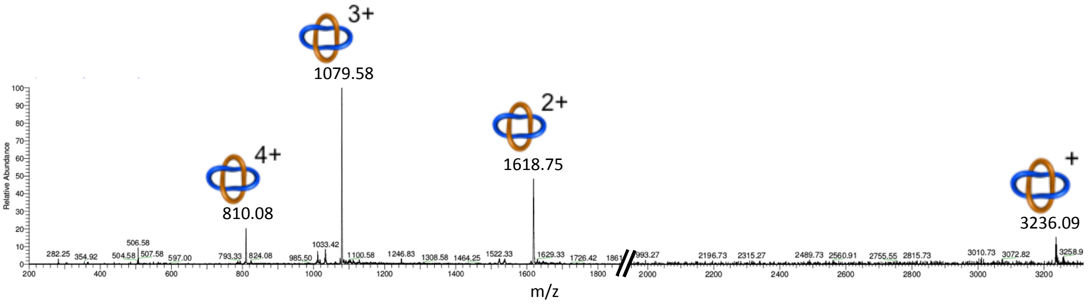

Figure S7 LRESI-MS analysis of **2**.

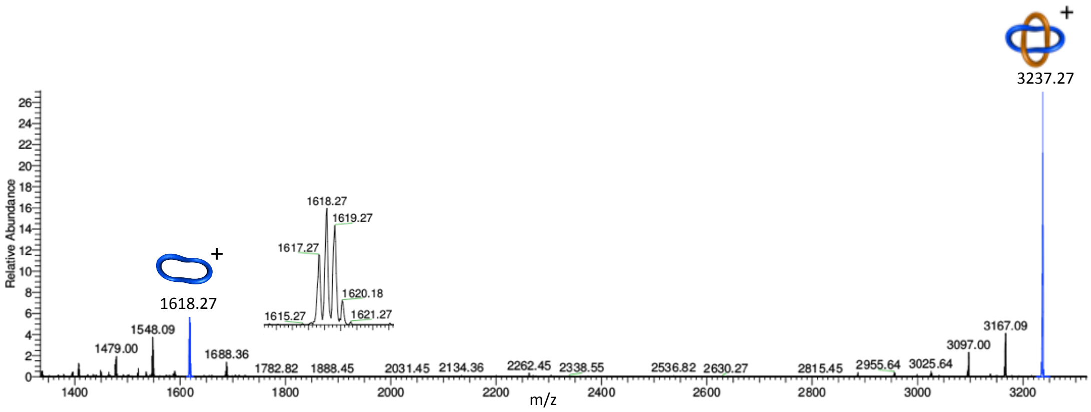

Figure S8 LRESI-MS^2 fragmentation of the [**2**•H]$^+$ ion. Major observed species are the original ion at 3237 m/z and the singly charged macrocycle at 1618 m/z, resulting from dethreading of the fragmented Solomon link. Each of these peaks is accompanied by a series of smaller signals corresponding to the loss of between one and three *iso*-pentyl groups.

3. X-ray Crystal Structure

Table S1. Summary of Crystal Data, Integrity Measurements and Structure Refinements for $[Zn_4\mathbf{2}](BF_4)_8$ complex.

$[Zn_4\mathbf{2}](BF_4)_8$	
**Crystal colour**	yellow
**Crystal size (mm)**	0.2 × 0.2 × 0.1
**Crystal system**	triclinic
**Space group, *Z***	2
a **(Å)**	14.142(2)
b **(Å)**	28.708(2)
c **(Å)**	32.942(2)
**α (°)**	96.348(6)
**β (°)**	102.250(8)
**γ (°)**	93.958(9)
**V (Å^3)**	12931(2)
**Density (Mg.m^{-3})**	0.990
**Wavelength (Å)**	0.6889
**Temperature (K)**	100
**μ(Mo-Kα) (mm^{-1})**	0.453
**2Θ range (°)**	3.066 to 33.362
**Reflns collected**	40565
**Independent reflns (R_{int})**	15663
**Reflns used in refinement, *n***	15663
**L.S. parameters, *p***	868
**No. of restraints, *r***	6737
**$R1$ (F),[a] $I > 2.0\sigma(I)$**	0.2541
**$wR2(F^2)$,[b] all data**	0.5746
**$S(F^2)$,[c] all data**	2.438

[a] $R1(F) = \Sigma(|F_o| - |F_c|)/\Sigma|F_o|$; [b] $wR^2(F^2) = [\Sigma w(F_o^2 - F_c^2)^2/\Sigma wF_o^4]^{½}$; [c] $S(F^2) = [\Sigma w(F_o^2 - F_c^2)^2/(n + r - p)]^{½}$

Data Collection. Synchrotron X-ray data were collected at beamline I19 (λ = 0.6889 Å) Diamond Light Source,[S2] for $[Zn_4\mathbf{2}](BF_4)_8$ at temperature of 100 K. Data were measured using CrystalClear-SM Expert 2.0 r5 suite of programs.

Crystal structure determinations and refinements. X-ray data were processed and reduced using the CrysAlisPro suite of programs. Absorption correction was performed using empirical methods based upon symmetry-equivalent reflections combined with measurements at different azimuthal angles.[S3] The crystal structure was solved and refined against all F^2 values using the SHELXTL suite of programs.[S4] Only zinc and sulfur atoms were refined anisotropically. The rest of the atoms were refined isotropically due to a poor reflexions/parameters ratio. Hydrogen atoms were placed in calculated positions refined using idealized geometries (riding model) and assigned fixed isotropic displacement parameters. The phenyl groups were restrained to have idealized geometries using AFIX commands. The C-C and C-O distances in the aliphatic chains, the B-F and F-F distances were restrained using DFIX and SADI command. The atomic displacement parameters (adp) of the ligands have been restrained using RIGU, EADP and SIMU commands.
Compound $[Zn_4\mathbf{2}](BF_4)_8$ present large voids filled with a lot of scattered electron density, the SQUEEZE protocol inside PLATON suites was used to account the void electron density.[S5]
A large number of A and B alerts were found due to poor crystal quality and data. Approximately all of these alerts (both A and B level) result from the large and deformed/ill-balanced thermal movement of the aliphatic chains. The cause of this effect can be attributed

to the unsolved disorder of these moieties. The high value of weighted R and the ratio of the observed/unique reflections, very large voids (residual electron density SQUEEZED) of the crystal lattice also give an A alert. The bond distance alerts result from the large thermal movement of the atoms.

The data had to be trimmed at 1.2 Å, because no diffraction was observed beyond. High weighted R factor and Rint are consequence of the poor crystal quality and data. As we can observe in Figure S6, Bragg diffraction spots were only observed in two directions of the space, while only diffuse scattering was found in the remaining direction. This phenomenon could be attributed to the disorder of the anions and the solvent molecules present in the inter-link region.

a) b)

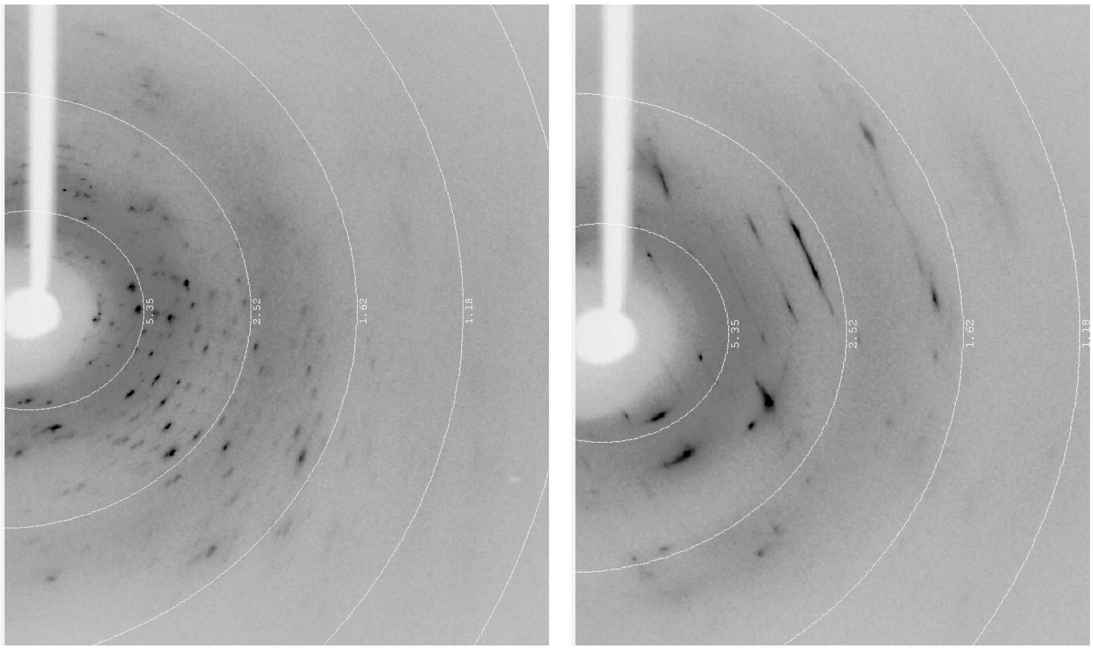

Figure S9 Diffraction images showing a) Bragg diffraction spots and b) diffusion scattering. Resolution arcs were added in white.

CCDC 1052083 contains the supplementary crystallographic data for this paper. These data can be obtained free of charge *via* www.ccdc.cam.ac.uk/conts/retrieving.html (or from the Cambridge Crystallographic Data Centre, 12 Union Road, Cambridge CB21EZ, UK; fax: (+44)1223-336-033; or deposit@ccdc.cam.ac.uk).

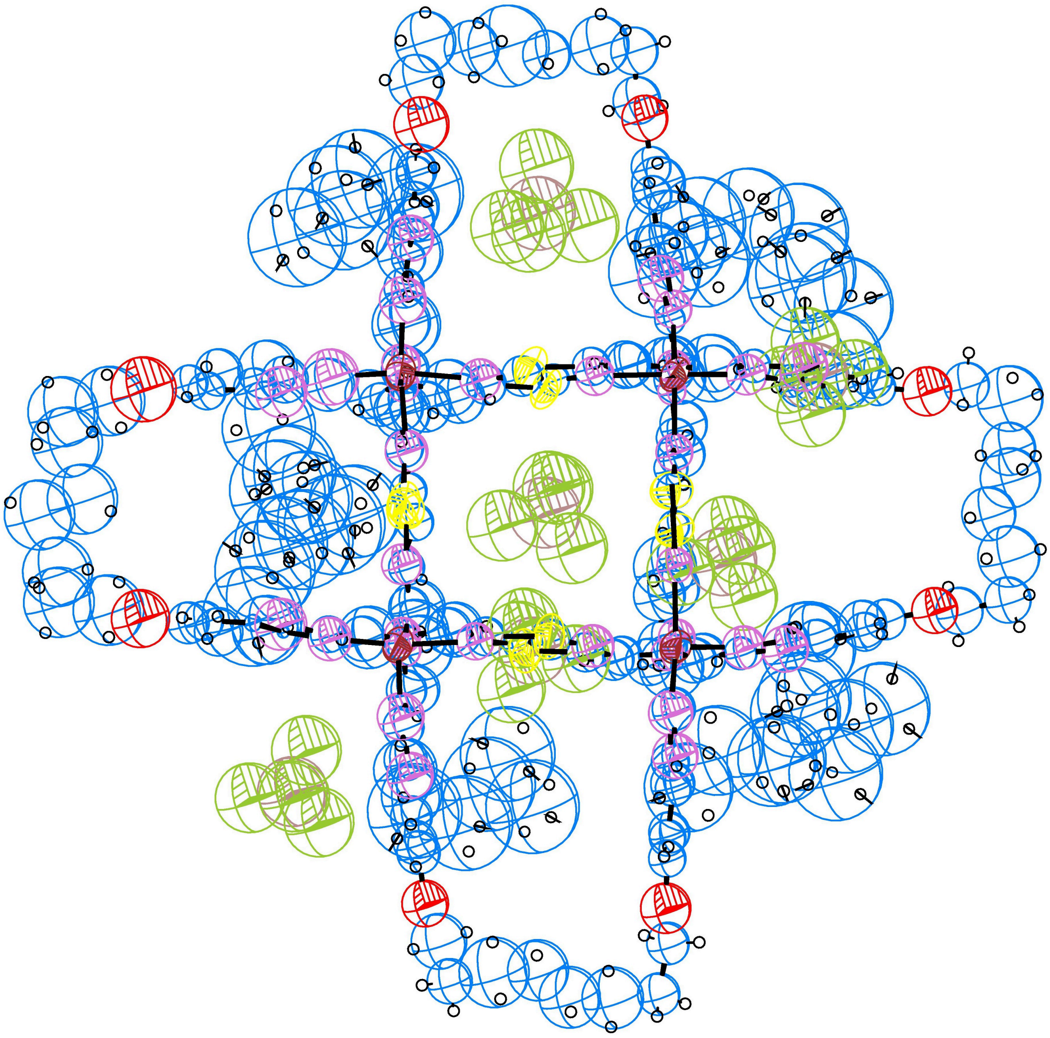

Figure S10 ORTEP representation of the X-ray crystal structure of $[Zn_4\mathbf{2}](BF_4)_8$ (ellipsoids at 50% probability). Only six of the eight BF_4^- anions are localized, including one in the center of the cavity of the grid.

(a)

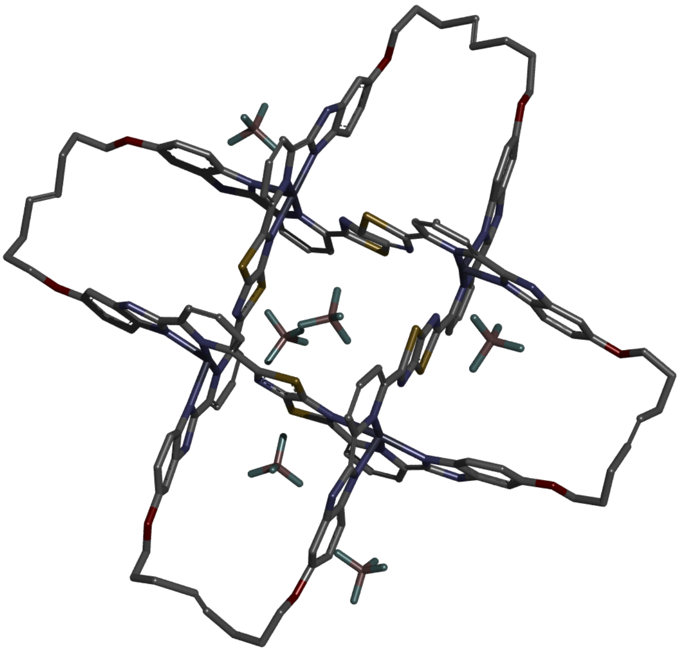

(b)

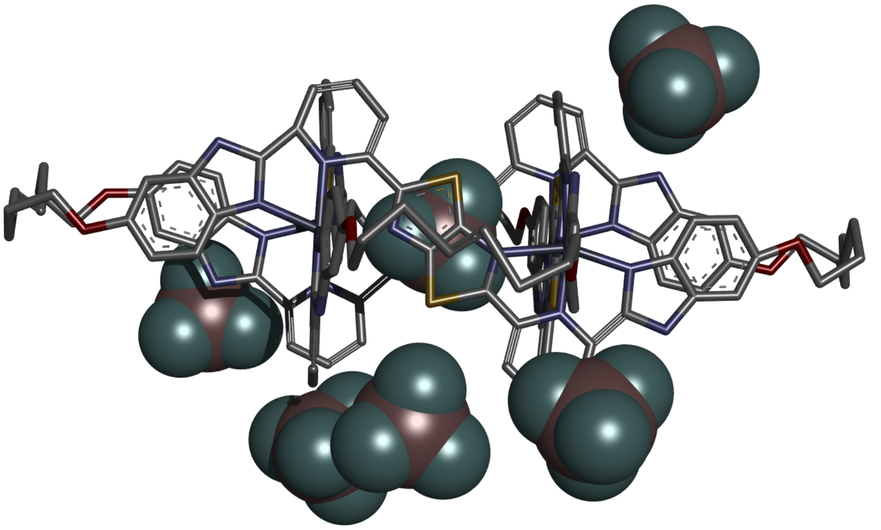

Figure S11 X-Ray crystal structure of $[Zn_4\textbf{2}](BF_4)_8$, including the six BF_4^- anions explicitly located in the structure. (a) Viewed from above the plane of the zinc ions. (b) viewed from the side.

4. Molecular modeling of Zn_4**2**

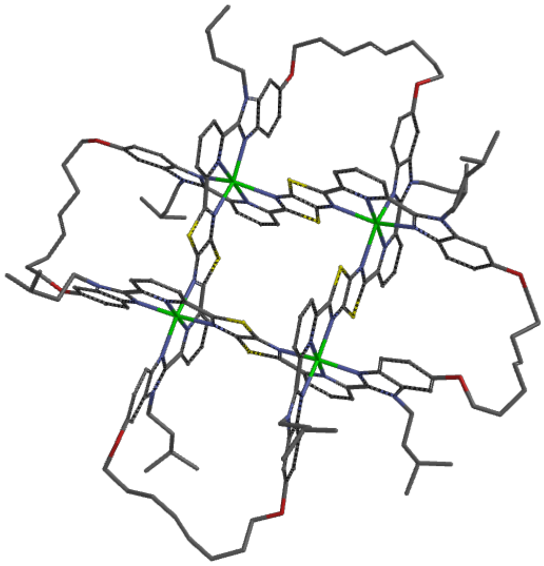

Figure S12 Modelled structure of Zn_4**2**. The geometry optimization was carried out using Spartan '14, Version 1.1.2 using MMFF minimization, and showed that the linker was of the necessary length to successfully achieve ring-closure between parallel strands.

5. Supporting Information References

[S1] R. Ziessel, P. Nguyen, L. Douce, M. Cesario, C. Estournes, *Org. Lett.* **2004**, *6*, 2865–2868.

[S2] H. Nowell, S. A. Barnett, K. E. Christensen, S. J. Teat, D. R. Allan, *J Synchrotron Radiat.* **2012,** *19*, 435–441.

[S3] a) G. M. Sheldrick, SADABS, Empirical absorption correction program based upon the method of Blessing, University of Göttingen, Göttingen (The Netherlands), **1995**; b) R. H. Blessing, *Acta Crystallogr.* **1995**, *A51*, 33–38.

[S4] a) G. M. Sheldrick, *Acta Crystallogr.* **2015**, *C71*, 3–8; b) G. M. Sheldrick, *Acta Crystallogr.* **2015**, *A71*, 3–8.

[S5] PLATON, A Multipurpose Crystallographic Tool, Utrecht University, Utrecht (The Netherlands), **2008**.
